# The Swiss data cube, analysis ready data archive using earth observations of Switzerland

**DOI:** 10.1038/s41597-021-01076-6

**Published:** 2021-11-08

**Authors:** Bruno Chatenoux, Jean-Philippe Richard, David Small, Claudia Roeoesli, Vladimir Wingate, Charlotte Poussin, Denisa Rodila, Pascal Peduzzi, Charlotte Steinmeier, Christian Ginzler, Achileas Psomas, Michael E. Schaepman, Gregory Giuliani

**Affiliations:** 1 University of Geneva, Institute for Environmental Sciences, GRID-Geneva, Bd. Carl-Vogt 66, Geneva, CH-1211 Switzerland; 2 University of Zurich, Remote Sensing Laboratories, Department of Geography, Winterthurerstrasse 190, Zurich, CH-8057 Switzerland; 3 Department F-A Forel for Aquatic and Environmental Sciences, Faculty of Sciences, University of Geneva, Geneva, Switzerland; 4 University of Geneva, Institute for Environmental Sciences, enviroSPACE Lab., Bd. Carl-Vogt 66, Geneva, CH-1211 Switzerland; 5 GRID-Geneva, Science Division, United Nations Environment Programme, 11, ch. Anémones, 1219 Geneva, Switzerland; 6 Swiss Federal Institute for Forest, Snow, and Landscape Research WSL, Zürcherstrasse 111, Birmensdorf, CH-8903 Switzerland

**Keywords:** Environmental sciences, Research data

## Abstract

Since the opening of Earth Observation (EO) archives (USGS/NASA Landsat and EC/ESA Sentinels), large collections of EO data are freely available, offering scientists new possibilities to better understand and quantify environmental changes. Fully exploiting these satellite EO data will require new approaches for their acquisition, management, distribution, and analysis. Given rapid environmental changes and the emergence of big data, innovative solutions are needed to support policy frameworks and related actions toward sustainable development. Here we present the Swiss Data Cube (SDC), unleashing the information power of Big Earth Data for monitoring the environment, providing Analysis Ready Data over the geographic extent of Switzerland since 1984, which is updated on a daily basis. Based on a cloud-computing platform allowing to access, visualize and analyse optical (Sentinel-2; Landsat 5, 7, 8) and radar (Sentinel-1) imagery, the SDC minimizes the time and knowledge required for environmental analyses, by offering consistent calibrated and spatially co-registered satellite observations. SDC derived analysis ready data supports generation of environmental information, allowing to inform a variety of environmental policies with unprecedented timeliness and quality.

## Abstract (summary)

**Table:** 

Measurement(s)	surface reflectance • backscatter
Technology Type(s)	satellite imaging
Sample Characteristic - Location	Switzerland

Machine-accessible metadata file describing the reported data: 10.6084/m9.figshare.14635485

## Background & Summary

Key environmental challenges arising from global change drivers (e.g., land use change, climate change, pollution, (over-)exploitation of resources, invasions) need to be overcome to manage the continuously growing pressures on countries’ natural resources. To address these key challenges and move towards sustainable development, we need to more rapidly provide relevant information on these drivers and their changes. This information can then be used to monitor environmental impacts in near-real time, and assess progress stemming from new policies (such as European directives or national policies), thereby allowing an evaluation of whether these changes have a positive or negative impact on these policies or if new, adapted policies may be formulated^1^. Earth Observation (EO) data from ground-based, airborne or satellite-borne instruments provide an effective way to monitor environmental changes.

Emerging global trends of (1) free and open data access policies for Landsat and Sentinel data; (2) the increasing provision of Analysis Ready Data (ARD) from EO satellites and (3) provision of open source software for managing and exploiting EO data, enables monitoring environmental changes at various spatial and temporal scales while complementing traditional data sources such as national statistics, administrative data or census information^1–3^. Significant work has recently been done to lower barriers and facilitate the access of end-users to harness the full potential of EO data, and to address mandates, national processes, or reporting obligations^4^. Earth Observation Data Cubes (EODCs) have emerged as a technology to manage, access and analyse Big EO Data, thereby strengthening connections between data providers, applications and end-users^5^.

Switzerland, alongside with Australia^6^, adopted this approach very early and provided an operational EODC, combined with EO Analysis Ready Data (ARD) covering the national territory. The Swiss Data Cube (SDC – http://www.swissdatacube.ch) is an initiative supported by the Swiss Federal Office for the Environment (FOEN) and developed, implemented and operated by the United Nations Environment Program (UNEP)/GRID-Geneva in partnership with the University of Geneva (UNIGE), the University of Zurich (UZH), and the Swiss Federal Institute for Forest, Snow and Landscape Research (WSL). It serves several purposes: (1) automated processing of satellite imagery to transform low level processed data into information products. (2) solving the conformity (i.e., standardization of measurements) issue of Earth Observation^7^, supporting Multilateral Environmental Agreements; and (3) allowing to test new methodologies for monitoring our environment.

In line with the above purposes, the SDC automatizes the generation of information products by providing easy access to, and tools to analyse, synoptic data facilitating research into such domains as climate, vegetation, agriculture, urban areas, water quality, snow coverage or generally all changes of our biotic, abiotic and human altered environment. Data access to complex data is facilitated by transforming them into a coherent and co-registered space-time format that is ready for analysis. In order to achieve conformity, the SDC allows product comparison and direct access to Swiss national *in-situ* sampling networks to validate the output^8^. Finally, SDC serves as a ‘sandbox’, where based on open-science principles information and design of new algorithms and methodologies for monitoring our environment is facilitated for all users. In addition, data producers are invited to ingest their data into the SDC, as well as adding new analytical methods, improving the possibilities of the analyses even further. Finally, the SDC provides substantial potential for the private sector, offering transparent, comprehensive and synoptic EO data for the purpose of self-evaluating carbon footprints or other environmental impact measures.

## Methods

### The swiss data cube

The Swiss Data Cube (SDC) initiative has started in 2016 under the mandate of the Swiss Federal Office for the Environment (FOEN). UNEP/GRID-Geneva and University of Geneva were mandated to explore the potential of the Data Cube technology initially developed in Australia^9^ to efficiently exploit freely and openly available satellite EO data; these are increasingly made freely available by agencies including the United States Geological Survey (USGS), the National Aeronautics and Space Agency (NASA) Landsat program, and the European Commission’s Copernicus and Sentinel programs^10,11^. After a successful initial phase where the SDC had been implemented by the GRID-Geneva and the University of Geneva, the University of Zurich/Remote Sensing Laboratories (UZH/RSL) and the Swiss Federal Institute for Forest, Snow and Landscape Research (WSL), joined the initiative to foster the use of EO data for environmental monitoring at national scale. The objective of the SDC is to deliver unique and near real-time capabilities to access and analyse EO data, enabling more effective responses to problems of national significance. It can provide the long-term and baseline data required to determine trends, quantify past and present changes, and inform future decisions. This near real-time information can be readily used as an evidence base for the design, implementation and evaluation of policies, programs and regulation, as well as for developing policy advice. Ultimately, it can support the Swiss government for environmental monitoring and reporting commitments, while enabling national scientific institutions to benefit from satellite EO data for research and innovation. Indeed, this technology is significantly improving the way non-expert users can work with EO data ready for analysis; for example, it reduces the time and scientific knowledge required to handle satellite imagery by automating the complex tasks of searching, downloading and pre-processing scenes, while at the same time facilitating the processing of large amount of satellite data^12^.

**Fig. 1:**
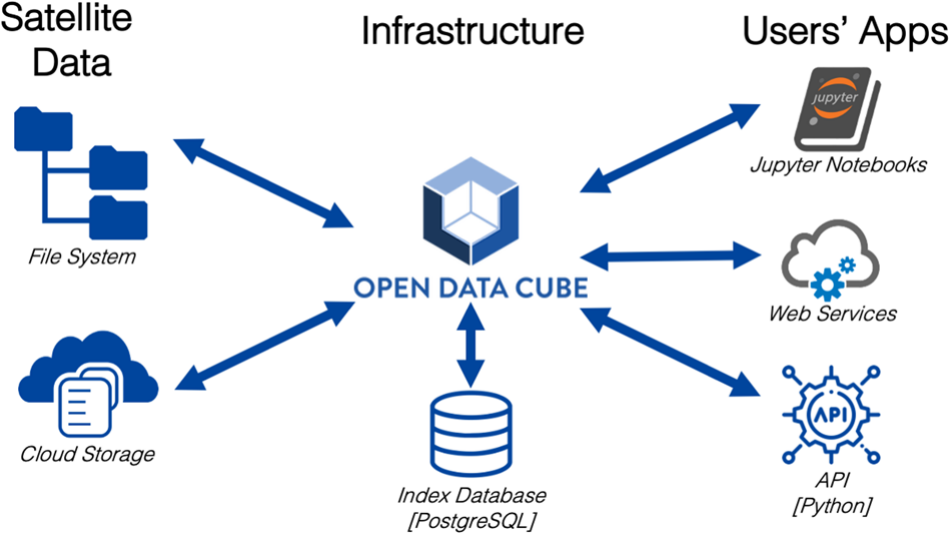
Swiss Data Cube general architecture and software components. (adapted from https://medium.com/opendatacube/what-is-open-data-cube-805af60820d7 and https://www.opendatacube.org/overview).

The SDC is built on the Open Data Cube (ODC – https://www.opendatacube.org) software suite. The ODC is an open source project, initiated by Geoscience Australia, the Commonwealth Scientific and Industrial Research Organization (CSIRO), the USGS, NASA and the Committee on Earth Observations Satellites (CEOS)^13^. It is designed to provide a framework to access, store, manage, and analyse large quantities of gridded satellite EO data collections. In particular, the ODC enables the cataloguing of large amounts of satellite EO data; it provides a Python-based Application Programming Interface (API) for data analysis and allows the tracking of data provenance for quality control and updates^14^ (Fig. 1).

The systematic and regular delivery of Analysis Ready Data (ARD)^15^ is fundamental to facilitate the generation of usable information products and supporting the development of end-users applications. CEOS defines ARD as *“satellite data that have been processed to a minimum set of requirements and organized into a form that allows immediate analysis with a minimum of additional user effort and interoperability both through time and with other datasets”*^16^. ARD reduces the burden of full utilization of satellite data by providing specifications that limits data preparation efforts to generate relevant, consistent, normalized, interoperable data. These specifications save time, efforts and minimizes the cost of pre-processing data while capitalizing on knowledge and expertise of users by allowing them to spend more time in analysing their data, rather than searching and pre-processing them. The requirements concern parameters such as radiometric and geometric calibration, atmospheric correction, and metadata descriptions^16^. In optical imagery, the ARD level corresponds to surface reflectance products^17^ whereas in radar imagery it corresponds to radiometrically normalised (terrain-flattened) backscatter^18^.

**Fig. 2:**
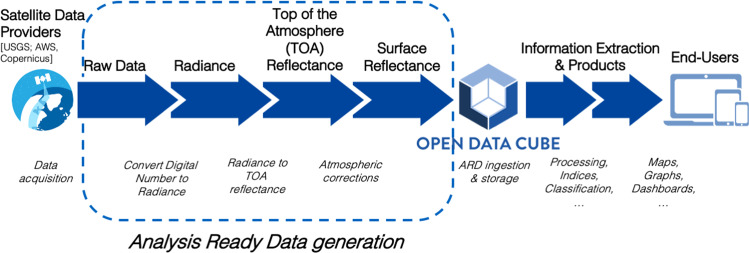
Common workflow for generation of optical Analysis Ready Data products.

Considering the fact that currently data providers such as the USGS Earth Resources Observation and Science (EROS) Center Science Processing Architecture (ESPA) (for Landsat data) and the Copernicus Open Access Hub (for Sentinel data) are not yet commonly generating ARD products (they usually deliver top of the atmosphere reflectance (L1C) while surface reflectance (L2A) archives are not yet complete), a fundamental element to consider is to have standardised and effective methods for generating ARD products and ensuring that all data ingested and stored in a data cube are consistent. Most of the steps of these procedures can be automated to search, download, and pre-process data for various data holdings, and managing different types of data (e.g., Landsat, Sentinel) to generate ARD products. To reach this objective, the Live Monitoring of Earth Surface (LiMES) framework^19^ has been used to generate Landsat 5,7,8^12^; Sentinel-1^20^; Sentinel-2^21^ ARD products that are stored in the SDC. LiMES is a framework based on composable chains of interoperable services for automated EO data discovery, access and (pre-)processing to convert EO data into environmental monitoring information products (Fig. 2).

### Data acquisition and analysis ready data products generation

The SDC holds Analysis Ready EO satellite Data since 1984 for the entire Switzerland. This archive, updated on a daily basis, contains (at the time of writing) 12’435 scenes corresponding to a total volume of 5 TB and more than 1000 billion observations/pixels. It stores data from the two largest EO data providers, the United States Landsat program and the European Copernicus Sentinel program^22,23^. Each satellite characteristics and ARD workflow are described hereafter. The temporal resolution (i.e., revisit time) for Landsat is 16 days, for Sentinel-1 is 6 days (12 per satellite, 6 in tandem)^24^ and for Sentinel-2 is 5 days (10 days per satellite, 5 in tandem)^25^.

**Landsat 5-7-8** are sun-synchronous satellites jointly operated by the USGS) and NASA^26,27^. They are essentially designed for land applications such as Earth resources, land surface, environmental monitoring, agriculture and forestry, disaster monitoring and assessment, ice and snow cover^28^. The Landsat 5 mission lasted from 1984 to 2013 and carried two sensors, the Multispectral Scanner System (MSS) and the Thematic Mapper (TM). Landsat 7 mission started in 1999 and will be decommissioned in 2021. The spacecraft carried the Enhanced Thematic Mapper (ETM+) sensor. The sensor’s Scan-Line Corrector (SLC) failed in July 2003^29^ and approximately 225 of the pixels per scene have since then not been scanned. However, the spatial and spectral quality of the remaining 78% of pixels images remain valid^30^. Landsat 8 was launched in 2013 and the mission is expected to continue until 2023^31–33^. A new generation of sensors were carried on Landsat 8, the Operational Land Imager (OLI) and Thermal Infrared Sensor (TIRS). Further details on these missions can be found in the CEOS Earth Observation Handbook for Landsat 5 (http://database.eohandbook.com/database/missionsummary.aspx?missionID=226), Landsat 7 (http://database.eohandbook.com/database/missionsummary.aspx?missionID=349), and Landsat 8 (http://database.eohandbook.com/database/missionsummary.aspx?missionID=547)^15,26,32,34^. The Landsat archive is the longest EO program, initiated in 1972, it has been providing continual and consistent observations for almost 50 years^32^. Since 2008, the complete data archive has been provided under a free and open access policy^35,36^. This has enabled dense time-series analysis, moving beyond simple diachronic comparison of a set of images, therefore dramatically improving capabilities to monitor environmental changes^37^. To cover the whole of Switzerland, it requires eight Landsat scenes (Path/Row: 193/027, 194/027, 195/027, 196/027, 193/028, 194/028, 195/028, 196/028) representing an area of latitude 44.9 to 48.7 and longitude 4.1 to 12.8. Data are downloaded as Collection 1/Tier 1 – Level 2 Surface Reflectance encompassing a surface of approximately 185 km by 180 km^15^. Collection 1/Tier 1 scenes are data with the highest available data quality (e.g., geometric and radiometric corrections) and considered suitable for time-series analysis^38^ (https://www.usgs.gov/land-resources/nli/landsat/landsat-collection-1). Level 2 corresponds to surface reflectance (i.e., the estimate based on Landsat sensor observations of the fraction of incoming solar radiation reflected from Earth’s surface). These data are corrected for atmospheric perturbations (e.g., aerosol scattering, thin clouds) enabling direct comparison between multiple images and dates. This corresponds to the ARD level. Two different models are applied to generate these ARD products. Landsat 5 and 7 TM are corrected with the Landsat Ecosystem Disturbance Adaptive Processing System (LEDAPS) algorithm^39^ whereas Landsat 8 OLI applies the Land Surface Reflectance Code (LaSRC) algorithm^40^. Both models use auxiliary climate data from MODIS and a radiative transfer model to evaluate atmospheric conditions over a given scene (https://www.usgs.gov/land-resources/nli/landsat/landsat-surface-reflectance).

As part of the SDC, a Python script has been implemented to search, download, and ingest Landsat data. This script generates a list of available and not yet ingested scenes for a given coverage and based on the scene ID, the required data are downloaded. Requests for Level 2 data are submitted via an API to the USGS Earth Resources Observation and Science (EROS) Center Science Processing Architecture (ESPA) On Demand Interface (https://espa.cr.usgs.gov). Once data are downloaded, they are directly ingested into the Swiss Data Cube, and a copy of the original data is kept as a backup. This workflow is updated on a weekly basis to ensure that the archive is always up to date. Currently, the number of ingested Landsat scenes correspond to 5643 images (L5: 2467 images; L7: 2146 images; L8: 1030 images), with a total volume of 1.3 TB (L5: 547 GB; L7: 429 GB; L8 347 GB) covering the years from 1984 up to the present day^41–43^. The yearly growth of the archive accounts for ~300 images and ~100 GB.

**Sentinel-1** (S1) is a constellation of currently two sun-synchronous satellites launched by the European Space Agency (ESA) in April 2014 (Sentinel-1A) and April 2016 (Sentinel-1B) respectively. Sentinel-1C and -1D are funded and will be launched after 2023. Each satellite carries a C-band Synthetic Aperture Radar (SAR) with 4 modes of operation. The most-commonly operated mode over land, and the one we used as input was the “interferometric wide-swath mode” (IW), which acquires data at VV and VH polarisations over Europe. The sensor is used e.g., to monitor sea ice zones and the arctic environment as well as marine environments. Given a set of single-look-complex (SLC) input data, they can be used to estimate land surface motion over short and long time-scales using synthetic aperture radar interferometry (InSAR). In our case, ground-range-detected (GRD) products were used as input, so retrievals based on the phase-difference were not possible. The satellites can provide mapping in support of humanitarian aid in crisis situations. A Sentinel-1 Next Generation (S1NG) is being planned for the 2030’s. Detailed descriptions of the sensors are provided in the CEOS EO Handbook for Sentinel-1A (http://database.eohandbook.com/database/missionsummary.aspx?missionID=575) and Sentinel-1B (http://database.eohandbook.com/database/missionsummary.aspx?missionID=576)^44^. To produce Sentinel-1 Analysis Ready Radiometrically Terrain Corrected (RTC) products, ground range detected (GRD) products were first downloaded from the EU open access source scihub.copernicus.eu and multilooking in the ground range and azimuth dimensions (10 looks in each case) was applied to reduce data volume and expedite processing. The Shuttle Radar Topography Mission (SRTM) digital elevation model (DEM) available at 3 arcsecond resolution was used for reference^45^. The DEM was used as an input to an image simulation of the local contributing area within each radar product image sample. That area was used to normalise the backscatter (rather than the ellipsoid model otherwise typically used) in ground range geometry. Then that normalised backscatter from each available polarisation (VV & VH) was terrain-geocoded (orthorectified) into the chosen map coordinates, producing a level-1 RTC product^46^. A comparison^19^ between products from the original UZH RTC software^39^ against later implementations of the same algorithm showed older editions of the ESA SNAP software introduced artefacts – this issue is being addressed by ESA with the SNAP software maintainers.

Sets of multiple RTC products (each holding VV & VH polarisations) acquired from different orbit tracks within a defined temporal window were then combined using local resolution weighting^47^ into level-3 multitemporal backscatter composite products. The region covered is latitude 44.5 to 48.5 N, longitude 5.5-11E, filling 20GB per year at 90 m spatial resolution and 6 day temporal spacing^48^. Higher spatial and temporal resolutions (e.g. Copernicus DEM at 30 m) are planned for the future.

**Sentinel-2** (S2) is a constellation of two sun-synchronous satellites operated by ESA respectively from 2015 to 2022 (Sentinel-2A) and 2017–2024 (Sentinel-2B)^25^. It supports land monitoring related services, including generation of generic land cover maps, risk mapping and fast images for disaster relief, generation of leaf coverage leaf chlorophyll content and leaf water content^49,50^. Both satellites carry the MultiSpectral Instrument (MSI) and further details can be found at: Sentinel-2 A (http://database.eohandbook.com/database/missionsummary.aspx?missionID=552) and Sentinel-2 B (http://database.eohandbook.com/database/missionsummary.aspx?missionID=553)^25,44^. They will be completed and replaced by Sentinel-2 C/D where launch is expected from 2021. Switzerland is covered by twelve Sentinel-2 scenes (ID: 31TGM, 31TGN, 32TLR, 32TLS, 32TLT, 32TMR, 32TMS, 32TMT, 32TNS, 32TNT, 32TPS, 32TPT). This corresponds to a spatial extent of latitude 45.0 to 47.9 N, longitude 5.5 to 11.8E. Data are downloaded as L1C products corresponding to 100 × 100 km^2^ geometrically corrected Top Of the Atmosphere (TOA) tiles^25,51^. To efficiently download, access, pre-process and ingest data, a python script has been developed to handle these different steps executed in nightly mode, and to make Sentinel-2 ARD available in the Swiss Data Cube. The first operation is to search and download data by querying the Copernicus Open Access Hub as well as Cloud storage facilities such as those provided by Amazon and Google. The implementation supports both the Open Data Protocol API (https://scihub.copernicus.eu/userguide/ODataAPI) and the gsutil tool (https://cloud.google.com/storage/docs/gsutil). The most efficient combination for S2 download speed, stability, storage, and readiness is to access data from the Google Cloud with data in Cloud Optimized GeoTIFF format (https://www.cogeo.org)^21^. It ensures good performance for data discovery (i.e., generating a list of available tiles) and access (i.e., fast download). Once data are downloaded, they enter a second step to correct the disturbances caused by the atmosphere and generate normalized surface reflectance that correspond to the ARD level (i.e., Level 2 A). For that, we use the ESA Sen2Cor algorithm (http://step.esa.int/main/third-party-plugins-2/sen2cor/)^52^ version 2.5.5 (2.8.0 version is not used as it is not able to process products before 2017). The algorithm follows two steps to pre-process data: first, it executes an image classification to identify and generate masks of clouds, cloud shadows, snow and water. Second, it applies an atmospheric correction model to convert TOA values into surface reflectance. Sen2Cor provides additional outputs on Aerosol Optical Thickness (AOT) and Water Vapour (WV)^53^. In the S2 ARD workflow, all bands are pre-processed and ingested except the band 10 that is only used for atmospheric corrections and consequently not relevant for users. It is important to mention that topographic correction is not applied as it creates strong artefacts; this can be seen by simply comparing Level 1 and Level 2 products of the same scene available on Google Cloud Platform (which applied topographic correction with sen2cor 2.8.0). Once data are pre-processed, they are then ingested into the Swiss Data Cube at a 20 m spatial resolution and are henceforward readily available for users. It should be noted that it has been decided to keep a copy of the original downloaded data because S2 data are stored in a rolling archive in the Copernicus Open Access Hub. This allows reprocessing the entire archive in case new atmospheric correction or more accurate topographic correction algorithms become available.

Finally, for users to fully benefit from the different satellite data types available in the Swiss Data Cube, we ensure that all observations (pixels and tiles) perfectly overlap. This means that one 30 m Landsat pixel covers exactly nine 10 m Sentinel-2 pixels without modification of data (e.g., resampling). This is done by extending the geographical extent in order to get latitude and longitude extent with finite multiple of Landsat and Sentinel-2 pixels and tiles. This is an important aspect to consider, allowing users to benefit from both sensors and to develop time-series analysis that uses multi-sensor data in a consistent way. This workflow is updated on a daily basis to ensure that the archive is always up to date. Currently, the number of S2 ingested tiles corresponds to 6792 images for a total volume of 3.6 TB covering the years from 2015 up to the present days^54^. The yearly growth of the archive accounts for 2000 images and 1 TB.

To ensure consistent and accurate provision of ARD, an important aspect to consider is the need to have a reprocessing strategy. Indeed, data providers can update their specifications (e.g., geometric, atmospheric corrections) and this may influence time-series analysis by breaking the consistency of data. Since the beginning the ARD generation workflow of the SDC is based on the LiMES framework providing a composable chain of interoperable services (Fig. 2)^19^. It is supported by large storage capacities and high-performance distributed computers providing a scalable, flexible and efficient processing environment for EO data. We have decided to keep a copy of the original data downloaded from the different repositories. One can argue that it duplicates data, but we consider that is important to have a full copy of the archive on our premises for reprocessing data. For example, if in the future we wish to use a different software component for atmospheric corrections, such as FORCE^55^ or MAJA^56^, we can easily replace the existing components in the pre-processing chain and then reprocess entirely the archive. Updated ARD (e.g., Landsat 5, Sentinel-2) or data products (e.g., NDVI time-series) are then possibly versioned to ensure the consistency of the different products and disclaimers/description are mentioned in the metadata description. With such an approach we seek to minimize the possible impacts to the wide variety of users’ expertise, many expecting error-free data. Moreover, as further described in the technical validation section, each datasets have quality information to help users to determine their suitability for specific applications.

## Data Records

The final dataset includes all Landsat 5-7-8, Sentinel-1, and Sentinel-2 processed at the Analysis Ready Data level. The five collections (Table 1) are freely available and accessible (for registered users) using the Python Application Programming Interface (API) at: http://sdc.unepgrid.ch:8080 or the web-based Graphical User Interface: http://sdc.unepgrid.ch.

**Table 1:** ARD collections description stored in the SDC.

Name	Platform	Instrument	Product Type	Measurements	Description	CRS	Format
s2_l2a_10m_swiss	SENTINEL-2	MSI	dc_preproc	coastal_aerosol, blue, green, red, veg5, veg6, veg7, nir, narrow_nir, water_vapour, swir1, swir2, slc	Standard surface reflectance related bands	EPSG:4326	NetCDF
ls5_ledaps_swiss	LANDSAT-5	TM	LEDAPS	blue, green, red, nir, swir1, swir2, pixel_qa, radsat_qa, cloud_qa	Standard surface reflectance related bands	EPSG:4326	NetCDF
ls7_ledaps_swiss	LANDSAT-7	ETM	LEDAPS	As Landsat 5	Standard surface reflectance related bands	EPSG:4326	NetCDF
ls8_lasrc_swiss	LANDSAT-8	OLI-TIRS	LaSRC	As Landsat 5	Standard surface reflectance related bands	EPSG:4326	NetCDF
s1_l3comp_swiss	SENTINEL_1_L3C	SAR	Gamma0	VV, VH	Radiometrically normalised (terrain-flattened) backscatter	EPSG:4326	NetCDF

A static copy of each collection is made available at the University of Geneva Research Data repository (https://yareta.unige.ch)^41–43,48,54^.

Each collection is described with according metadata following the ISO19115 standard and description are available at: https://geonetwork.swissdatacube.org

Landsat 5: https://geonetwork.swissdatacube.org/geonetwork/srv/eng/catalog.search?node=srv#/metadata/4dc0defe-68c3-4546-8608-33c5c8351c11

Landsat 7: https://geonetwork.swissdatacube.org/geonetwork/srv/eng/catalog.search?node=srv#/metadata/f475c001-1bee-4b53-9a70-dd34a80f29cf

Landsat 8: https://geonetwork.swissdatacube.org/geonetwork/srv/eng/catalog.search?node=srv#/metadata/e1ad9b5d-2287-4cd0-9b89-08ab4cf627f6

Sentinel-1: https://geonetwork.swissdatacube.org/geonetwork/srv/eng/catalog.search?node=srv#/metadata/a7f13bda-7df4-40ec-a059-73bf21553eb3

Sentinel-2: https://geonetwork.swissdatacube.org/geonetwork/srv/eng/catalog.search?node=srv#/metadata/fbc005c9-9168-47af-beb2-0862e8325622

In addition, metadata and data are available as Open Geospatial Consortium (OGC) standards web services endpoints to ensure interoperable discovery, visualization and download.

- Metadata Discovery - Catalog Service for the Web (CSW 2.0.2): https://geonetwork.swissdatacube.org/geonetwork/srv/eng/csw?request=GetCapabilities&service = CSW&version = 2.0.2
- Data visualization - Web Map Service (WMS 1.3.0): http://sdc.unepgrid.ch/ows/wms?request=GetCapabilities&service=WMS&version = 1.3.0 and Web Map Tile Service (WMTS 1.0.0): http://sdc.unepgrid.ch/ows/wmts?request=GetCapabilities&service=WMTS&version=1.0.0
- Data download – Web Coverage Service (WCS 1.0.0,2.0.0,2.1.0): http://sdc.unepgrid.ch/ows/wcs?request=GetCapabilities&service=WCS&version=2.1.0

**Fig. 3:**
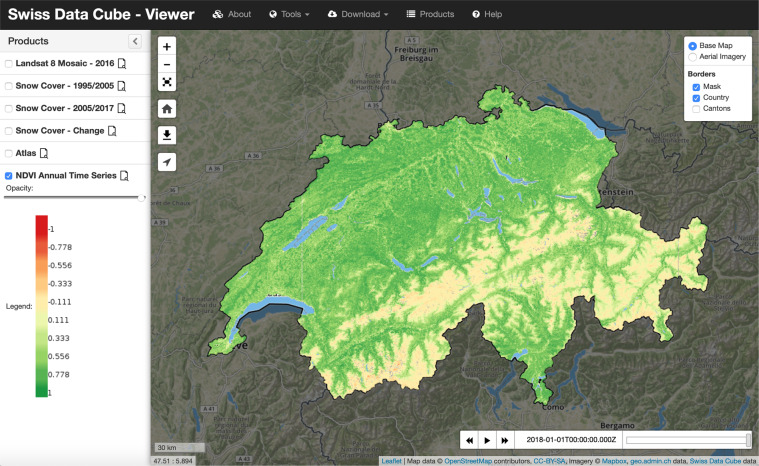
An example of SDC data visualized in the Swiss Data Cube Viewer.

These endpoints enable users to access and/or integrate these datasets in their desktop, web-based clients (Fig. 3) or own specific analysis workflows.

The Swiss Data Cube can be easily updated with other satellite EO data collections (e.g., MODIS, Sentinel-5P). As new data will be organized and pre-processed following the protocols presented in this paper, new data steams can be readily included.

## Technical Validation

All downloaded data are checked by a data curator before being pre-processed and ingested. Each satellite data scene/tile should be documented through specific metadata that provides sufficient information for pre-processing and ingestion.

**Landsat** data are provided by USGS with Quality Assessment (QA) information to help users to determine their suitability for specific applications. An 8-bit LandsatLook Quality Image and 16-bit Quality Assessment Band^57^ are also included. Details on each file are described at: https://www.usgs.gov/land-resources/nli/landsat/landsat-collection-1-level-1-quality-assessment-band. Level 2 products are generated by the USGS from level 1 product and using the official LEDAPS/LASRC algorithm. “A Pixel Quality Assurance (pixel_qa) band is provided with all Landsat Surface Reflectance-derived Spectral Indices. The band is in unsigned 16-bit format, values are bit-packed and provide information pertaining to a pixel condition of fill, clear, water, cloud shadow, snow, cloud (yes/no), cloud confidence and cirrus cloud confidence (Landsat 8 only)” https://www.usgs.gov/land-resources/nli/landsat/landsat-sr-derived-spectral-indices-pixel-quality-band.

**Sentinel-1**: The geometry of Sentinel-1 data is well calibrated: data can be geocoded without any tiepoints to an accuracy of a few centimetres^58,59^, far better than is possible with optical sensors. Sentinel-1 radiometric stability is monitored and calibrated within the Sentinel-1 Mission Performance Centre^60^. A review of the quality of multiple implementations of radiometric terrain correction (RTC) processing was published in 2019^20^. The quality of the terrain correction and radiometric corrections depends on the quality of the input DEM made available. Participants in the Copernicus programme (currently not including Switzerland) are able to access a world-wide high-quality DEM with 30 m resolution, with 10 m models available in some regions.

**Sentinel-2** processed to level 2 A with Sen2Cor comes with a scene classification (similar to the pixel_qa band provided by USGS for Landsat data) with 12 classes as described at: https://earth.esa.int/web/sentinel/technical-guides/sentinel-2-msi/level-2a/algorithm. It helps identifying pixels that are saturated or defective as well as cloud and cloud shadows that may affect the quality of the images. In addition, ESA provides a monthly status of the quality of Sentinel-2 data through Data Quality Reports (DQR). These reports document geometric and radiometric performances against initial sensor specifications together with observed anomalies and issues. DQR are available at: https://sentinels.copernicus.eu/web/sentinel/data-product-quality-reports

## Usage Notes

The Analysis Ready Data provided by the Swiss Data Cube (SDC) contributes to provide information that are synoptic, consistent and spatially explicit, and therefore ideal to monitor environmental changes across the country. These can be provided at pixel level, or aggregated at various administrative levels (communes, cantons or national scale). It can contribute to national policies^1,61^, support Sustainable Development Goals^62,63^, and monitor land changes^64,65^ (e.g., snow, agriculture, urban, biodiversity, water quality,…). Ultimately, *in-situ* measurements (e.g., on-the-ground sensors) are essential and should be used in conjunction to calibrate and validate generated remotely-sensed data products.

Currently, the SDC is funded on a project-based approach; in the near future, however, a more sustainable funding mechanism is called for, one possibility being through a support for a national digital infrastructure. To reach this objective, several steps are envisioned to demonstrate the effectiveness of the SDC. The first phase is completed as researchers from UNEP/GRID-Geneva, University of Geneva, University of Zurich and the Swiss Federal Institute for Forest, Snow and Landscape (WSL) are able to use this technology for environmental monitoring in Switzerland. In a second phase, we will encourage more beneficiaries at the Federal, Cantonal and Communal level to test the SDC as an information service for decision makers. We believe that a collaborative, open-science approach^66^, freely accessible to everyone from both the public and private sectors, will greatly pay back all investments and exceed them by providing validated information services. Researchers are also supporting other countries in developing data cube technology and could (accounting for the funds and IT infrastructure) provide data cubes on demand to any location in the world at short notice. Finally, researchers are planning to extend this information service and make it compatible with other data sources, such as cadastral, statistical and meteorological data. The system will provide interfaces to other existing infrastructures. Currently, the approach is based on freely available data such as Landsat and Sentinel. However, the SDC is able to ingest different types of geospatial data provided by various sources… Alternative data cubes on meteorological data, on cadastral and statistical (socio-economic indicators) could be designed by other partners, offering the possibility to cross these data cubes for new types of analysis. This could create a national base digital infrastructure, with significant gain in production, easy analysis, and allow envisioning developing digital replicas of different aspects of the Swiss natural systems (i.e. digital twins).

For researchers, data preparations (i.e., download, stacking the bands, various corrections) are time consuming processes with limited scientific interest. SDC provides “Analysis Ready Data” by automatizing the image processing to a level where the real scientific analysis can start. It removes the “tedious tasks” so that scientists can concentrate on the real value added, i.e., the design of algorithms for retrieving indices, image classifications, trend and time series analysis, environmental assessments, change detections and associated scientific analysis. It offers centralized access to several sensors (so far Landsat, Sentinel-1 and Sentinel-2) all pre-processed using best standards. The quality is therefore granted and homogeneous, ensuring comparability between data. Furthermore, it also allows for the fusion between e.g., radar and passive optical sensor imagery. Finally, the development (on-going) of a solid IT infrastructure based on parallel computing will allow several computing methodologies to be tested. Currently, the main focus in this direction is to port the SDC platform on the High-Performance Computing (HPC) environment available at UNIGE (Baobab cluster - https://baobabmaster.unige.ch/enduser/enduser.html). This will allow different users to work in parallel and have their own individual development environment. The second phase of this initiative is to go even further and improve the processing performances by parallelizing the SDC not only at the data level but also at algorithms level, using specialized parallelization libraries, such as DASK (https://dask.org/).

SDC can provide the basis for environmental protection, which has societal, economic and ecological consequences (e.g. erosion/degradation of agricultural areas), it has implications also in economy, ecology (e.g. biodiversity^67^), food production and security. Importantly, this technology can be used for quickly assessing the impacts of policies on the ground (e.g., water quality, urban development and agricultural policies) and for production of indices (i.e., drought, vegetation and snow). SDC can be used to support decisions on land planning, and for assessing trends to infer on futures scenarios. Climate change and pressures on ecosystems require timely decisions based on science. SDC provides a complete coverage of the territory and is frequently updated. It is ideal for providing rapid monitoring^68^ on many topics and it is cost efficient, as most of the transformation of data to information is automatized.

SDC generates the baseline for the service industry to grow. A condition for this growth is reliable and validated data and algorithms from an independent, but highly trustworthy source. SDC could be used as a tool for estimating the impact of environmental damage or environmental hazards on infrastructures in a coherent way and at a national level. This would be beneficial for the (re-)insurance sector and other financial services such as commodity trading, or more generally, every private sector which is affected by environmental issues or relying on earth observational data. Other estimation areas: economic impacts on the agricultural sector, food production and forecasting of the impact of environmental change to potentially reconsider current practices (e.g., water management), funding of start-up ventures, which depend on the trend in snow coverage for example in the building of new ski resorts, etc.

SDC implements available tools to make data discovery, access and processing as interoperable as possible^69^. It implements all relevant standards promoted by the Open Geospatial Consortium (OGC) such as the Web Map Service (WMS) for data visualization; Web Coverage Service (WCS), Catalog Service for the Web (CSW) and ISO19139 for metadata; and Web Processing Service (WPS) for processing. It will ultimately comply with the FAIR data principles to make data Findable, Accessible, Interoperable and Reusable^70,71^. This will help to contribute to relevant initiative such the Digital Switzerland^72^ or the Global Earth Observation System of Systems^73^ to support decisions and actions through coordinated, comprehensive and sustained EO data and information^74^.

## Data Availability

The SDC is based on the Open Data Cube platform that can be obtained at: https://www.opendatacube.org and freely available under an Apache 2.0 license (https://opensource.org/licenses/Apache-2.0). The Analysis Ready Data workflows are developed as a suite of Python (3.6) and R (2.7) scripts. Both programming languages are freely available at: https://www.python.org and https://cran.r-project.org respectively under Python Software Foundation License (PSFL) and GNU General Public License. Landsat, Sentinel-1, Sentinel-2 workflows are freely available on the Swiss Data Cube GitHub repository https://github.com/GRIDgva/SwissDataCube/tree/master/ingestors under GNU GPL 3.0 license (https://www.gnu.org/licenses/gpl-3.0.en.html). The Landsat Ecosystem Disturbance Adaptive Processing System (LEDAPS) (version 3.4.0) is an atmospheric correction model developed by USGS. The repository was recently removed from Github and is being re-established on the USGS Official Source Code Archive https://code.usgs.gov/espa and will be soon freely available under Unlicense conditions (https://www.usgs.gov/core-science-systems/nli/landsat/espa-and-product-related-code-repository-location-changes). Land Surface Reflectance Code (LaSRC) (version 1.4.1) is an atmospheric correction model developed by USGS. The repository was recently removed from Github and is being re-established on the USGS Official Source Code Archive https://code.usgs.gov/espa and will be soon freely available under Unlicense conditions (https://www.usgs.gov/core-science-systems/nli/landsat/espa-and-product-related-code-repository-location-changes). Sen2cor (version2.5) is a processor for Sentinel-2 Level 2 A product generation and formatting. It is freely available at: https://step.esa.int/main/third-party-plugins-2/sen2cor/ under an Apache 2.0 license. The Sentinel-1 processing is performed using UZH’s in-house developed radiometric image simulation and geocoding software radsim v2.0, currently undergoing packaging for wider distribution. More information can be obtained by contacting Dr. David Small (University of Zurich): https://www.geo.uzh.ch/geolean/en/department/Staff/?content=davidsmall. However, the algorithm used to generate Sentinel-1 analysis ready radiometrically terrain-corrected (RTC) Synthetic Aperture Radar (SAR) gamma nought backscatter data described in^46^ is also available in the European Space Agency (ESA) Sentinel Application Platform (SNAP) that is freely available at: http://step.esa.int/main/download/snap-download/ under a GNU GPL v3 license. GeoNetwork is a web-based application (version 3.10.5) used for cataloguing, publishing and managing metadata description of spatially referenced resources using OGC and ISO standards. It is freely available at: https://geonetwork-opensource.org under a GNU General Public License v2.0. Datacube-ows has been used to expose data collections according to OGC standards for visualization (Web Map Service – WMS) and download (Web Coverage Service – WCS). It is freely available at: https://github.com/opendatacube/datacube-ows under an Apache 2.0 license.

## References

[1] Dhu T (2019). National Open Data Cubes and Their Contribution to Country-Level Development Policies and Practices. Data.

[2] Harris R, Baumann I (2015). Open data policies and satellite Earth observation. Space Policy.

[3] Doldirina, C. Open Data and Earth Observations - The Case of Opening Up Access to and Use of Earth Observation Data Through the Global Earth Observation System of Systems. *jipitec* **6** (2015).

[4] Wu, B., Tian, F., Zhang, M., Zeng, H. & Zeng, Y. Cloud services with big data provide a solution for monitoring and tracking sustainable development goals. *Geography and Sustainability* S2666683920300109 (2020).

[5] Gomes VCF, Queiroz GR, Ferreira KR (2020). An Overview of Platforms for Big Earth Observation Data Management and Analysis. Remote Sensing.

[6] Lewis A (2016). Rapid, high-resolution detection of environmental change over continental scales from satellite data - the Earth Observation Data Cube. Int J Digit Earth.

[7] Widlowski J-L (2015). Conformity testing of satellite-derived quantitative surface variables. Environmental Science & Policy.

[8] Braun D, Damm A, Hein L, Petchey OL, Schaepman ME (2018). Spatio-temporal trends and trade-offs in ecosystem services: An Earth observation based assessment for Switzerland between 2004 and 2014. Ecological Indicators.

[9] Lewis A (2017). The Australian Geoscience Data Cube — Foundations and lessons learned. Remote Sensing of Environment.

[10] European Commission, *Commission Delegated Regulation (EU) No 1159/2013 of 12 July 2013 supplementing Regulation (EU) No 911/2010 of the European Parliament and of the Council on the European Earth monitoring programme (GMES) by establishing registration and licensing conditions for GMES users and defining criteria for restricting access to GMES dedicated data and GMES service information Text with EEA relevance*. *OJ L* vol. 309 (2013).

[11] Ryan, B. The benefits from open data are immense. *Geospatial World* 72–73 (2016).

[12] Giuliani G (2017). Building an Earth Observations Data Cube: lessons learned from the Swiss Data Cube (SDC) on generating Analysis Ready Data (ARD). Big Earth Data.

[13] Killough, B. Overview of the Open Data Cube Initiative. in *IGARSS 2018 - 2018 IEEE International Geoscience and Remote Sensing Symposium* 8629–8632, 10.1109/IGARSS.2018.8517694 (2018).

[14] Rizvi, S. R., Killough, B., Cherry, A. & Gowda, S. The Ceos Data Cube Portal: a User-Friendly, Open Source Software Solution for the Distribution, Exploration, Analysis, and Visualization of Analysis Ready Data. in *IGARSS 2018 - 2018 IEEE International Geoscience and Remote Sensing Symposium* 8639–8642, 10.1109/IGARSS.2018.8518727 (2018).

[15] Dwyer J (2018). Analysis Ready Data: Enabling Analysis of the Landsat Archive. Remote Sensing.

[16] Lewis, A. *et al*. CEOS Analysis Ready Data for Land (CARD4L) Overview. in *IGARSS 2018 - 2018 IEEE International Geoscience and Remote Sensing Symposium* 7407–7410, 10.1109/IGARSS.2018.8519255 (2018).

[17] CEOS. Product Family Specification Optical Surface Reflectance (CARD4L-OSR). (2019).

[18] CEOS. Product Family Specification: Normalised Radar Backscatter. (2019).

[19] Giuliani G (2017). Live Monitoring of Earth Surface (LiMES): A framework for monitoring environmental changes from Earth Observations. Remote Sensing of Environment.

[20] Truckenbrodt J (2019). Towards Sentinel-1 SAR Analysis-Ready Data: A Best Practices Assessment on Preparing Backscatter Data for the Cube. Data.

[21] Giuliani, G., Chatenoux, B., Honeck, E. & Richard, J. Towards Sentinel-2 Analysis Ready Data: a Swiss Data Cube Perspective. in *IGARSS 2018 - 2018 IEEE International Geoscience and Remote Sensing Symposium* 8659–8662, 10.1109/IGARSS.2018.8517954 (2018).

[22] Aschbacher J, Milagro-Pérez MP (2012). The European Earth monitoring (GMES) programme: Status and perspectives. Remote Sensing of Environment.

[23] Berger M, Aschbacher J (2012). Preface: The Sentinel missions—new opportunities for science. Remote Sensing of Environment.

[24] Torres R (2012). GMES Sentinel-1 mission. Remote Sensing of Environment.

[25] Drusch M (2012). Sentinel-2: ESA’s Optical High-Resolution Mission for GMES Operational Services. Remote Sensing of Environment.

[26] Egorov AV (2019). Landsat 4, 5 and 7 (1982 to 2017) Analysis Ready Data (ARD) Observation Coverage over the Conterminous United States and Implications for Terrestrial Monitoring. Remote Sensing.

[27] Wulder MA (2016). The global Landsat archive: Status, consolidation, and direction. Remote Sensing of Environment.

[28] Ustin SL, Middleton EM (2021). Current and near-term advances in Earth observation for ecological applications. Ecol Process.

[29] Chen J, Zhu X, Vogelmann JE, Gao F, Jin S (2011). A simple and effective method for filling gaps in Landsat ETM+ SLC-off images. Remote Sensing of Environment.

[30] Wulder MA, Ortlepp SM, White JC, Maxwell S (2008). Evaluation of Landsat-7 SLC-off image products for forest change detection. Canadian Journal of Remote Sensing.

[31] Wulder MA (2008). Landsat continuity: Issues and opportunities for land cover monitoring. Remote Sens Environ.

[32] Wulder MA (2019). Current status of Landsat program, science, and applications. Remote Sensing of Environment.

[33] Irons JR, Dwyer JL, Barsi JA (2012). The next Landsat satellite: The Landsat Data Continuity Mission. Remote Sensing of Environment.

[34] Arvidson T, Gasch J, Goward SN (2001). Landsat 7’s long-term acquisition plan — an innovative approach to building a global imagery archive. Remote Sensing of Environment.

[35] Wulder MA, Masek JG, Cohen WB, Loveland TR, Woodcock CE (2012). Opening the archive: How free data has enabled the science and monitoring promise of Landsat. Remote Sensing of Environment.

[36] Zhu Z (2019). Benefits of the free and open Landsat data policy. Remote Sensing of Environment.

[37] Giuliani G, Chatenoux B, Piller T, Moser F, Lacroix P (2020). Data Cube on Demand (DCoD): Generating an earth observation Data Cube anywhere in the world. International Journal of Applied Earth Observation and Geoinformation.

[38] Zhu Z (2019). Science of Landsat Analysis Ready Data. Remote Sensing.

[39] Masek JG (2006). A Landsat surface reflectance dataset for North America, 1990–2000. IEEE Geoscience and Remote Sensing Letters.

[40] Vermote E, Justice C, Claverie M, Franch B (2016). Preliminary analysis of the performance of the Landsat 8/OLI land surface reflectance product. Remote Sensing of Environment.

[41] Giuliani G, Chatenoux B, Richard J-P (2020). Yareta.

[42] Giuliani G, Chatenoux B, Richard J-P (2020). Yareta.

[43] Giuliani G, Chatenoux B, Richard J-P (2020). Yareta.

[44] Malenovský Z (2012). Sentinels for science: Potential of Sentinel-1, -2, and -3 missions for scientific observations of ocean, cryosphere, and land. Remote Sensing of Environment.

[45] Jarvis, A., Guevara, E., Reuter, H. I. & Nelson, A. D. *Hole-filled SRTM for the globe: version 4: data grid*. (2008).

[46] Small D (2011). Flattening Gamma: Radiometric Terrain Correction for SAR Imagery. IEEE Transactions on Geoscience and Remote Sensing.

[47] Small, D., Rohner, C., Miranda, N., Rüetschi, M. & Schaepman, M. E. Wide-Area Analysis-Ready Radar Backscatter Composites. *IEEE Transactions on Geoscience and Remote Sensing* 1–14 (2021).

[48] Giuliani G, Chatenoux B, Richard J-P, Small D (2020). Yareta.

[49] Gascon F (2017). Copernicus Sentinel-2A Calibration and Products Validation Status. Remote Sensing.

[50] Muller-Wilm, U., Louis, J., Richter, R., Gascon, F. & Niezette, M. Sentinel-2 Level 2A Prototype Processor: Architecture, Algorithms And First Results. in vol. 722, 98 (2013).

[51] Djamai N, Fernandes R (2018). Comparison of SNAP-Derived Sentinel-2A L2A Product to ESA Product over Europe. Remote Sensing.

[52] Louis, J. *et al*. SENTINEL-2 SEN2COR: L2A Processor for Users. In *Proceedings Living Planet Symposium* 2016 (ed. Ouwehand, L.) vol. SP-740 1–8 (Spacebooks Online, 2016).

[53] Main-Knorn, M. *et al*. Sen2Cor for Sentinel-2. In *Image and Signal Processing for Remote Sensing XXIII* vol. 10427, 1042704 (International Society for Optics and Photonics, 2017).

[54] Giuliani G, Chatenoux B, Richard J-P (2020). Yareta.

[55] Frantz D (2019). FORCE—Landsat + Sentinel-2 Analysis Ready Data and Beyond. Remote Sensing.

[56] Baetens L, Desjardins C, Hagolle O (2019). Validation of Copernicus Sentinel-2 Cloud Masks Obtained from MAJA, Sen2Cor, and FMask Processors Using Reference Cloud Masks Generated with a Supervised Active Learning Procedure. Remote Sensing.

[57] Ernst S (2018). Implications of Pixel Quality Flags on the Observation Density of a Continental Landsat Archive. Remote Sensing.

[58] Schubert A, Miranda N, Geudtner D, Small D (2017). Sentinel-1A/B Combined Product Geolocation Accuracy. Remote Sensing.

[59] Piantanida, R. *et al*. Accurate Geometric Calibration of Sentinel-1 Data. In *EUSAR 2018; 12th European Conference on Synthetic Aperture Radar* 1–6 (2018).

[60] Hajduch, G. *et al*. *The Sentinel-1 Mission Performance Center Activities and Support for the End Users Community*. 10.13140/RG.2.2.24577.51041 (2019).

[61] Asmaryan S (2019). Paving the Way towards an Armenian Data Cube. Data.

[62] Honeck E (2018). From a Vegetation Index to a Sustainable Development Goal Indicator: Forest Trend Monitoring Using Three Decades of Earth Observations across Switzerland. ISPRS International Journal of Geo-Information.

[63] Giuliani G (2020). Monitoring land degradation at national level using satellite Earth Observation time-series data to support SDG15 – exploring the potential of data cube. Big Earth Data.

[64] Poussin C (2019). Snow Cover Evolution in the Gran Paradiso National Park, Italian Alps, Using the Earth Observation Data Cube. Data.

[65] Salzano R (2019). Automated Classification of Terrestrial Images: The Contribution to the Remote Sensing of Snow Cover. Geosciences.

[66] Giuliani G, Camara G, Killough B, Minchin S (2019). Earth Observation Open Science: Enhancing Reproducible Science Using Data Cubes. Data.

[67] Randin CF (2020). Monitoring biodiversity in the Anthropocene using remote sensing in species distribution models. Remote Sensing of Environment.

[68] Giuliani G (2020). Essential Variables for Environmental Monitoring: What are the Possible Contributions of Earth Observation Data Cubes?. Data.

[69] Giuliani G, Masó J, Mazzetti P, Nativi S, Zabala A (2019). Paving the Way to Increased Interoperability of Earth Observations Data Cubes. Data.

[70] Stall S (2019). Make scientific data FAIR. Nature.

[71] Wilkinson MD (2016). The FAIR Guiding Principles for scientific data management and stewardship. Scientific Data.

[72] Federal Office of Communicatons OFCOM. Digital Switzerland Strategy.

[73] Craglia M, Hradec J, Nativi S, Santoro M (2017). Exploring the depths of the global earth observation system of systems. Big Earth Data.

[74] Giuliani G (2021). SwissEnvEO: A FAIR National Environmental Data Repository for Earth Observation Open Science. Data Science Journal.

